# 

**Published:** 1917-02-03

**Authors:** 


### CONTENTS.

| | PAGE |
|---|---|
| **The Report of the Medical Officer to the Local Government Board** ... ... ... | 355 |
| **Sanatorium Troubles** ... ... ... ... ... ... | 356 |
| **Hospital and Institutional News** ... ... ... | 357 |
| More Gigantic Legacies for Hospitals—Lord Rhondda and Quack Practice—The French System of Care for the Disabled Wounded—The Renewal and Maintenance of Artificial Limbs—The National Relief Fund and Hospitals—How Will They Spend the Money?—War Charities and Church Finance—A Large-Minded Colliery Company—An Opportune Reorganisation Scheme—Friction at Beverley—The Suppression of Deceptive Advertisements—Two Important Points as to Compulsory Segregation—Municipal Hospitals—"Economy" Over Health at Merthyr—The Consumption of Wine in the French Army—Type-Reading for the Blind—Is a Collector Worth His Salt?—The Collector Versus the Trained Secretary—The Sunday Shave in Hospitals—The Sanitary Inspector's Salary—Baby Saving in Wales—The Mentally Defective at Plymouth—Milk Sterilisation Criticised at Liverpool—The "Cocaine and Opium Regulations" and Institutions—Get Good Stocks of Clinical Thermometers—Analysis of Drugs—This Week's Drug Market—To Our Readers. | |
| **New Ideas in Obstetrics from America** ... | 363 |
| Current Views on Cæsarean Section. | |
| **The Drug and Alcohol Habit** ... ... ... ... | 364 |
| The Treatment of Delirium Tremens. | |
| **The Institutional Library** ... ... ... ... | 365 |
| Fire Prevention in Institutions. | |
| **The Dreadnought and the Explosion** ... ... | 367 |
| **The Treatment and Control of Venereal Diseases** ... ... ... ... ... ... ... | 367 |
| From the Standpoint of the Voluntary Hospitals. | |
| **More About the Royal British College of Nursing** ... ... ... ... ... ... | 369 |
| The Charge of Lay Control Demonstrably a Myth. | |
| Round the Hospitals. | |
| The Lady Possesses Lay Control—Recognition of Homeland Workers—Royal Red Cross Reform—An Empire Decoration—The Meeting in Dublin—Supplementary Register for Children's Nurses. | |
| **The Royal British College of Nursing** ... ... | 372 |
| Meeting in Dublin. | |
| **The Editor's Letter-Box** ... ... ... ... ... | 373 |
| Be Ready for Emergencies—The Explosion on January 19. | |
| **The Leicester Royal Infirmary Meeting** ... | 373 |
| The Depreciation of Institutional Investments. | |
| **London County and Westminster Bank, Limited** ... ... ... ... ... ... | viii |
| The War Loan: A Banker's Warning. | |
| **The Institutional Worker** ... ... (Suppl.) | 1 |
| Attendance Record for Casualty Cases: Answer to January Question. | |
| The Ward Linen Supply: Answer to January Question. | |

